# The immunoregulatory function of peripheral blood CD71^+^ erythroid cells in systemic-onset juvenile idiopathic arthritis

**DOI:** 10.1038/s41598-021-93831-3

**Published:** 2021-07-13

**Authors:** Hikaru Kanemasa, Masataka Ishimura, Katsuhide Eguchi, Tamami Tanaka, Etsuro Nanishi, Akira Shiraishi, Motohiro Goto, Yoshitomo Motomura, Shouichi Ohga

**Affiliations:** Departments of Pediatrics, Graduate School of Medical Sciences, Kyushu University, 3-1-1 Maidashi, Higashi-ku, Fukuoka, 812-8582 Japan

**Keywords:** Juvenile idiopathic arthritis, Erythropoiesis, Acute inflammation, Paediatric research

## Abstract

CD71^+^ erythroid cells (CECs) are recognized to have an immunoregulatory function via direct cell–cell interaction and soluble mediators. Circulating CECs appear in newborns or patients with hemolytic and cardiopulmonary disorders. To assess the biological role of CECs in systemic inflammation, we studied the gene expression and function in systemic-onset juvenile idiopathic arthritis (SoJIA). Peripheral blood mononuclear cells of SoJIA patients expressed upregulated erythropoiesis-related genes. It represented the largest expansion of CECs during active phase SoJIA among other inflammatory diseases. Despite the opposing roles of erythropoietin and hepcidin in erythropoiesis, both serum levels were in concert with the amounts of SoJIA-driven CECs. Circulating CECs counts in inflammatory diseases were positively correlated with the levels of C-reactive protein, IL-6, IL-18, or soluble TNF receptors. Co-culture with active SoJIA-driven CECs suppressed secretions of IL-1β, IL-6, and IL-8 from healthy donor monocytes. The top upregulated gene in SoJIA-driven CECs was *ARG2* compared with CECs from cord blood controls, although cytokine production from monocytes was suppressed by co-culture, even with an arginase inhibitor. CECs are driven to the periphery during the acute phase of SoJIA at higher levels than other inflammatory diseases. Circulating CECs may control excessive inflammation via the immunoregulatory pathways, partly involving arginase-2.

## Introduction

CD71^+^ erythroid cells (CECs) appear in umbilical cord blood or peripheral blood of newborns^1–3^. The numbers of nucleated red blood cells peak at birth in the periphery (approximately 500/μL) and then decline, ultimately disappearing by the fourth day of life^4^. CECs thus cannot be found in the peripheral blood of healthy infants, children, or adults. Peripheral blood CECs (PBCECs) have been observed in pathological settings of hematological disorders^5^, solid tumors^6^, acute respiratory distress syndrome^7^, cardiovascular diseases^8^, and sepsis^9^. Many studies have mentioned the link between increased numbers of PBCECs and serious conditions or poor prognoses^5–9^.

Red blood cells transport oxygen to the tissues and circulate in contact with complements in the plasma. Nucleated erythroid cells in newborn mice exert an immunosuppressive effect via soluble factors, including arginase-2, transforming growth factor-β, and reactive oxygen species^1,6,10^. Human erythrocytes modulate the innate immunity to bind and scavenge chemokines, cell-free nucleic acids, and pathogens in circulation^11^. CECs control the T cell function by direct cell–cell interaction via programmed death-ligand 1 (PDL-1) in both humans and mice^12^. We and other groups demonstrated the suppressive function of human cord blood CECs^13,14^, and others reported similar effects of PBCECs from patients with late-stage tumors^6^ or human immunodeficiency virus (HIV) infection^15^.

Systemic-onset juvenile idiopathic arthritis (SoJIA) is an autoinflammatory disorder of unknown etiology, characterized by spiking fever, lymphadenopathy, and generalized skin rash. Although it has been classified into one of the seven JIA subtypes^16^, the pediatric counterpart to adult-onset Still’s disease is considered a prototypic polygenic autoinflammatory disease distinct from other variants of JIA^17^. Affected children present with a high fever complicated by cytokine storm and then develop idiopathic arthritis during the course of the disease. Comprehensive genetic analyses of blood leukocytes and affected tissues have helped clarify the molecular mechanisms underlying autoimmunity and autoinflammation^18^. IL-1-related genes are highly expressed in healthy peripheral blood mononuclear cells (PBMCs) cultured with the patients’ sera^19^. IL-1-targeted therapy has been introduced as standard therapy for SoJIA^20,21^. The overproduction of IL-1 is a hallmark of autoinflammatory diseases arising from mutations in inflammasome-related genes, including *NLRP3*, *NLRC4*, *PSTPIP1*, *MEFV*, and *MVK*^22^. However, no evidence has been produced for monogenic diseases involving the IL-1 and IL-18 pathways or any environmental factors associated with the development of SoJIA.

Previous studies have documented the upregulation of genes related to erythropoiesis in PBMCs from patients with SoJIA^23–25^. Similar gene upregulations in PBMCs were observed in patients with autoinflammatory diseases such as cryopyrin-associated periodic syndrome^26^ and rheumatic arthritis^27^, or hypoxic disorders, including RS virus infection^28^, chronic obstructive pulmonary disease^29^, idiopathic pulmonary fibrosis^30^, and pulmonary hypertension^31^. The erythropoiesis-related genes are limitedly expressed in erythroid precursor cells but not leukocytes. The upregulation of erythropoiesis-related genes may be explained by a response to hypoxia, whereas its association with autoinflammatory diseases has never been explained. We have thus hypothesized that the upregulation of erythropoiesis-related genes arises from aberrant erythroblastosis in the setting of inflammatory disorders.

In the present study, we determined the upregulation of heme and hemoglobin-related genes in PBMCs from SoJIA patients and confirmed the expansion of PBCECs in patients with SoJIA or Kawasaki disease (KD). The co-culture with PBCECs from SoJIA patients suppressed the production of inflammatory cytokines from monocytes. We also discuss the biological role of PBCECs with a potential immunosuppressive function in inflammatory diseases.

## Results

### The upregulated expression of erythroid-related genes in PBMCs obtained from SoJIA patients

**Figure 1:**
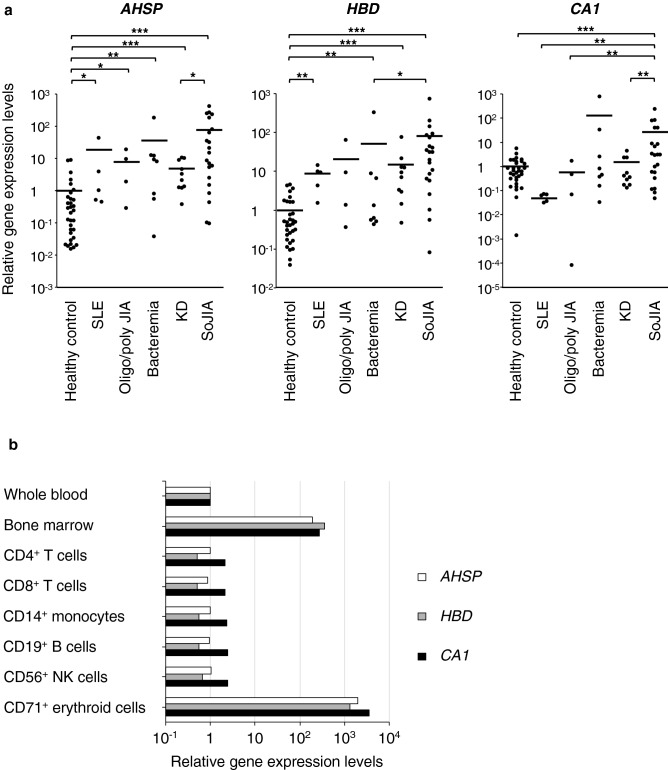
Genes derived from erythroid cells are highly expressed in SoJIA patients. (**a**) The gene expression of *AHSP*, *HBD,* and *CA1* in peripheral blood mononuclear cells. Cells are obtained from patients with systemic lupus erythematosus, oligo/polyarticular juvenile idiopathic arthritis, bacteremia, Kawasaki disease, and systemic-onset juvenile idiopathic arthritis as well as healthy donors. (**b**) The mRNA levels of *AHSP*, *HBD*, and *CA1* in whole blood and bone marrow mononuclear cells, along with purified populations of peripheral lymphocytes, monocytes, and CD71^+^ erythroid cells. Gene expression data are retrieved from the BioGPS database (http://biogps.org). The line indicates the average. Data are analyzed by the Mann–Whitney *U* test with Bonferroni correction. *P < 0.05; **P < 0.01; ***P < 0.001. *SLE* systemic lupus erythematosus, *Oligo/poly JIA* oligo/polyarticular juvenile idiopathic arthritis, *KD* Kawasaki disease, *SoJIA* systemic-onset juvenile idiopathic arthritis.

In a previous study^32^, we conducted a whole-genome microarray analysis of PBMCs among patients with SoJIA (n = 5), KD (n = 3), histiocytic necrotizing lymphadenitis (n = 4) and a healthy control to clarify the differences in the gene expression profile. The analysis identified 39 upregulated genes with a more than twofold difference in expression between patients with SoJIA and KD or histiocytic necrotizing lymphadenitis (Supplementary Table S1 online). The most strongly upregulated gene was *AHSP*, which encodes an erythroid-specific molecular chaperone that stabilizes a globin. Hemoglobin delta (*HBD*), the third-most strongly upregulated gene was also related to hemoglobin production, and *CA1*, the seventh-most strongly upregulated gene, encodes carbonic anhydrase 1, one of the markers of erythroid differentiation and a co-expression partner for HBD genes. Quantitative polymerase chain reaction (PCR) validation showed that the amounts of *AHSP*, *HBD*, and *CA1* gene transcripts were larger in patients with SoJIA than in healthy controls and those with KD, bacteremia, oligo/polyarticular JIA, and systemic lupus erythematosus (Fig. 1a). According to the BioGPS database (http://biogps.org), the *AHSP*, *HBD*, and *CA1* genes were overwhelmingly more strongly expressed in the bone marrow and CECs than in peripheral whole blood (Fig. 1b). These data suggested that CECs, not lymphocytes or monocytes, are the main source of heme/hemoglobin-related genes, and PBMCs from SoJIA patients contain abundant amounts of erythroid precursor cells.

### Expansion of CD71^+^ erythroid cells in PBMCs of SoJIA patients

**Figure 2:**
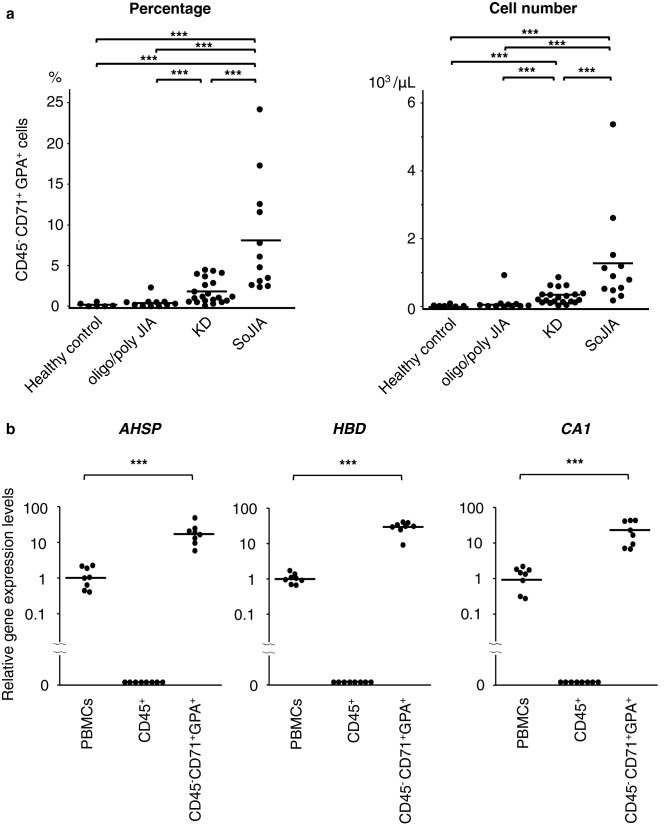
The expansion of CD45^−^ CD71^+^ GPA^+^ erythroid cells in peripheral blood mononuclear cells derived from patients with pediatric inflammatory diseases. (**a**) The percentage and absolute number of CD45^−^ CD71^+^ GPA^+^ erythroid cells in peripheral blood mononuclear cells among healthy control and patients with oligo/polyarticular JIA, KD, and SoJIA. (**b**) Gene expression of *AHSP*, *HBD*, and *CA1* in whole peripheral blood mononuclear cells, CD45^+^ lymphocytes and monocytes, and CD71^+^ erythroid cells. The CD45^+^ lymphocytes and monocytes and the CD71^+^ erythroid cells are selectively extracted from fresh peripheral blood mononuclear cells derived from SoJIA patients using a cell sorter. The line indicates the average. Data are analyzed by the Mann–Whitney *U* test with Bonferroni correction. *P < 0.05; **P < 0.01; ***P < 0.001. *oligo/poly JIA* oligo/polyarticular juvenile idiopathic arthritis, *KD* Kawasaki disease, *SoJIA* systemic-onset juvenile idiopathic arthritis.

Glycophorin A (GPA; also known as CD235a) is one of the most common markers of the erythroid lineage and CD71, a transferrin receptor, is a cell surface marker of all erythroid precursors, but mature erythrocytes lack its expression^33^. CD36 is a determinant marker of erythroid progenitors expressed continuously on normal immature erythroblasts, and its expression decreases gradually with their maturation^33^. CD71^+^ erythroid cells are not found in peripheral blood of healthy subjects. We therefore prospectively compared the numbers of CD71^+^ GPA^+^ erythroid cells among PBMCs obtained from patients with SoJIA, KD, oligo/polyarticular JIA, and healthy controls. Nucleated blood cells, including CD71^+^ erythroid cells, were isolated using density gradient centrifugation. Blood samples were obtained at the acute phase of SoJIA, KD, and oligo/polyarticular JIA. Fresh PBMCs were immediately separated to prevent any contamination with mature erythrocytes and assayed using a flow cytometry analysis. PBMCs from SoJIA patients showed redness, indicating the presence of more red blood cells than in others (data not shown). Flow cytometry demonstrated increased numbers and proportions of CD45^−^ CD71^+^ GPA^+^ erythroid cells in PBMCs from patients with SoJIA compared with those from patients with KD or oligo/polyarticular JIA (Fig. 2a and Supplementary Fig. S1a online). We focused on the assessment of CD45^+^ CD71^+^ GPA^+^ CECs, as a previous study demonstrated that CD45^+^ CECs contributed more strongly to the immunosuppressive function than their CD45^−^ counterparts^2,34^. Peripheral blood CD71^+^ GPA^+^ CECs were exclusively found in CD45-negative cell populations in our cohort subjects (Supplementary Fig. S1b online). The negligible amounts of CD45-positive CD71^+^ GPA^+^ CECs might account for the different conditions of patients including their age, type of illness, and duration of inflammation. Increased numbers and proportions of more immature erythroid cells (CD45^−^ CD36^+^ CD71^+^ GPA^+^ cells) were also detected in patients with SoJIA compared with patients with KD or oligo/polyarticular JIA (Supplementary Fig. S1c,d online). Using cell-sorting techniques, we confirmed that the *AHSP*, *HBD*, and *CA1* transcriptions were increased in CD45^−^ CD71^+^ GPA^+^ erythroid cells but not in CD45^+^ lymphocytes or monocytes (Fig. 2b). These data supported the notion that the heme/hemoglobin-related genes highly expressed in PBMCs from SoJIA patients originated from PBCECs.

### Erythroid cells are decreased among PBMCs in the convalescent phase of SoJIA

**Figure 3:**
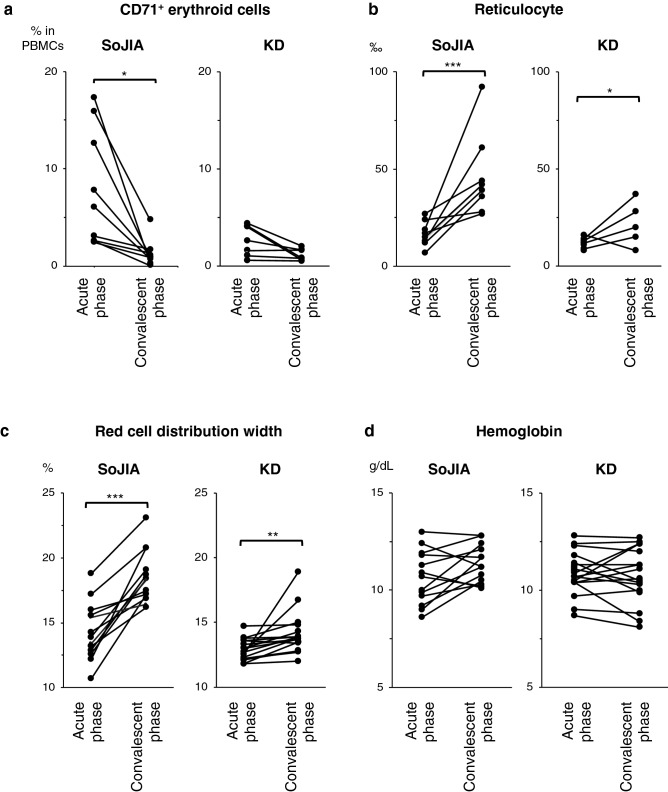
Emergence of CD71^+^ erythroid cells in the peripheral blood in the acute febrile phase of the disease. (**a**–**d**) The comparison of the (**a**) percentage of peripheral CD71^+^ erythroid cells, (**b**) permille of the reticulocytes, (**c**) red cell distribution width, and (**d**) hemoglobin levels between the acute febrile phase and convalescent phase. Data are collected during the pretreatment acute febrile phase and the convalescent phase after effective treatment when the C-reactive protein drops to < 0.5 mg/dL. The line indicates the average. Data are analyzed by the Mann–Whitney *U* test with Bonferroni correction. *P < 0.05; **P < 0.01; ***P < 0.001. *SoJIA* systemic-onset juvenile idiopathic arthritis, *KD* Kawasaki disease, *RDW* red cell distribution width.

To determine the association between CECs and inflammation, we compared the number and proportion of PBCECs between the acute febrile phase and the convalescent (afebrile and negative C-reactive protein [CRP]) phase. PBCECs decreased after defervescence (Fig. 3a). In contrast, the percentage of reticulocytes and red cell distribution width (RDW) increased after effective therapy (Fig. 3b,c). We used the reticulocyte count and RDW as markers for the result of erythropoiesis. A previous study also demonstrated the high RDW in the convalescence of SoJIA^25^. There was no significant change in the hemoglobin levels (Fig. 3d). These trends in erythropoiesis-related indexes were observed in patients with SoJIA or KD but were particularly augmented in SoJIA.

### Factors related to the emergence of erythroid cells in peripheral blood

We hypothesized that the excessive inflammation induced the introduction of CECs into the peripheral blood, as patients with KD or SoJIA showed higher levels of inflammatory markers than those with oligo/polyarticular JIA, such as an increased white blood cell count and elevated serum levels of CRP, ferritin, IL-6, IL-18, sTNFR1, and sTNFR2 (Table 1). Among these markers, the levels of ferritin, IL-18, and sTNFR1 were significantly higher in patients with SoJIA than in those with KD (*P* = 0.046, 0.001, and 0.03, respectively).

**Table 1:** Clinical variables at the sample collection for the detection of PBCECs using flow cytometry.

	1. Oligo/poly JIA	2. Kawasaki disease	3. Systemic-onset JIA	*P* value		
				1 vs. 2	1 vs. 3	2 vs. 3
Number of patients	11	21	12			
Age (years)	8, 2–16	3, 0.3–9	7, 2–13	0.009	0.27	0.09
Male :female	5:6	13:8	5:7			
**Laboratory data**						
White blood cells (10^9^/L)	7.1, 5.3–9.4	11.9, 2.9–20.8	26.7, 7.2–33.8	0.01	0.004	0.14
Hemoglobin (g/dL)	12.6, 10.9–14.6	11.2, 8.7–13.5	11.8, 8.4–14.9	0.14	0.36	1.0
Red cell distribution width (%)	12.8, 11.6–15.3	12.6, 11.8–13.8	14.5, 11.6–16.2	1.0	0.06	0.01
Reticulocyte^b^ (/μL)	0.07, 0.06–0.11	0.05, 0.04–0.09	0.07, 0.03–0.13	0.07	1.0	1.0
C-reactive protein (mg/dL)	0.3, 0.02–3.7	5.4, 2.2–9.5	10.2, 0.02–36.0	< 0.001	0.01	0.37
Ferritin (ng/mL)	34, 4–127	105, 44–1,101	333, 60–12,662	0.002	0.002	0.046
Erythropoietin^c^ (mIU/mL)	4, 2–36	20, 0–30	38, 13–123	1.0	0.65	0.47
Hepcidin^c^ (pg/mL)	31, 0–57	239, 99–501	574, 283–630	0.006	< 0.001	0.009
IL-6^a^ (10^3^ pg/mL)	0, 0–0	24, 0–112	53, 0–1098	0.002	0.002	0.74
IL-18^a^ (ng/mL)	46, 13–292	188, 44–697	3143, 396–21,140	0.008	< 0.001	< 0.001
IL-18BP^a^ (pg/mL)	686, 594–866	1310, 647–9236	1950, 667–3427	0.14	0.28	1.0
sTNFR1^a^ (mIU/mL)	267, 15–314	404, 86–885	675, 194–1213	0.06	< 0.001	0.03
sTNFR2^a^ (pg/mL)	3033, 1830–3606	7371, 3201–12,688	7113, 3041–14,951	0.001	0.002	1.0
IFN-γ^a^ (ng/mL)	19, 0–40	24, 0–262	10, 0–69	1.0	1.0	0.22
G-CSF^a^ (mIU/mL)	13, 0–28	23, 0–62	15, 0–209	1.0	0.60	1.0
GM-CSF^a^ (pg/mL)	6, 0–14	6, 0–11	4, 0–11	1.0	0.67	0.52
Galectin-3^a^ (ng/mL)	25, 9–34	35, 12–85	67, 21–186	0.22	0.008	0.03
Duration of fever^d^ (days)	–	5.5, 1–11	7, 2–22	–	–	0.55

Data are shown as the number and percentage or median and range. Bonferroni adjustment was used for multiple comparisons. Comparative analyses were performed among patients with data available. Patients with oligo/polyarticular JIA vs. Kawasaki disease vs. systemic-onset JIA.

*JIA* juvenile idiopathic arthritis, *IL-18BP* Interleukin-18 binding protein, *sTNFR* soluble tumor necrosis factor receptor, *IFN-γ* interferon-gamma.

^a^10 vs. 14 vs. 9.

^b^9 vs. 13 vs. 7.

^c^3 vs. 7 vs. 5.

^d^0 vs. 21 vs. 12.

To identify the factors that induced circulating erythroid progenitors, we assessed the correlation between the percentage of erythroid cells in PBMCs and biomarkers related to inflammation, hematopoiesis, and erythropoiesis (Table 2 and Supplementary Fig. S2 online). Serum levels of erythropoietin and hepcidin, leading to erythropoiesis or secondary anemia, respectively, showed a positive correlation with the amounts of PBCECs (r = 0.67 and 0.64, respectively). Serum hepcidin but not erythropoietin levels were significantly higher in patients with SoJIA than in those with KD (Table 1). Endogenous G-CSF levels also had a positive correlation with PBCECs (r = 0.49) (Table 2), and the levels did not differ markedly between SoJIA and KD patients (Table 1). These findings suggested a factor contributing to the expansion of PBCECs in SoJIA other than hematopoiesis enhanced by erythropoietin and G-CSF. The PBCEC count was correlated with the white blood cell count and serum levels of IL-6, CRP, IL-18, and sTNFR1 (r = 0.61, 0.55, 0.55, 0.42, and 0.37, respectively), indicating an association with systemic inflammation (Table 2). Among these variables, the IL-18 and sTNFR1 levels were significantly higher in SoJIA patients than in KD patients or other controls (Table 1). These results indicate the association between the magnitude of inflammation and the expansion of PBCECs.

**Table 2:** The variables showing significant correlation with the percentage of PBCECs by the Pearson’s correlation analysis.

	Correlation coefficients, r	*P* value
Erythropoietin^b^ (mIU/mL)	0.67	0.009
Hepcidin^b^ (pg/mL)	0.64	0.003
White blood cell count^a^	0.61	< 0.001
Interleukin-6^a^ (pg/mL)	0.55	< 0.001
C-reactive protein^a^ (mg/dL)	0.55	< 0.001
G-CSF* (mIU/mL)	0.49	0.003
Red cell distribution width^a^ (%)	0.45	0.01
IL-18^a^ (ng/mL)	0.42	0.02
sTNFR1^a^ (mIU/mL)	0.37	0.03

There were no variables showing significant negative correlation with the percentage of PBCECs. The number of analyzed patients with oligo/polyarticular JIA, Kawasaki disease, and systemic-onset JIA.

^a^10, 14, and 9.

^b^3, 7, and 5.

### The immunoregulatory effect of PBCECs obtained from SoJIA patients

**Figure 4:**
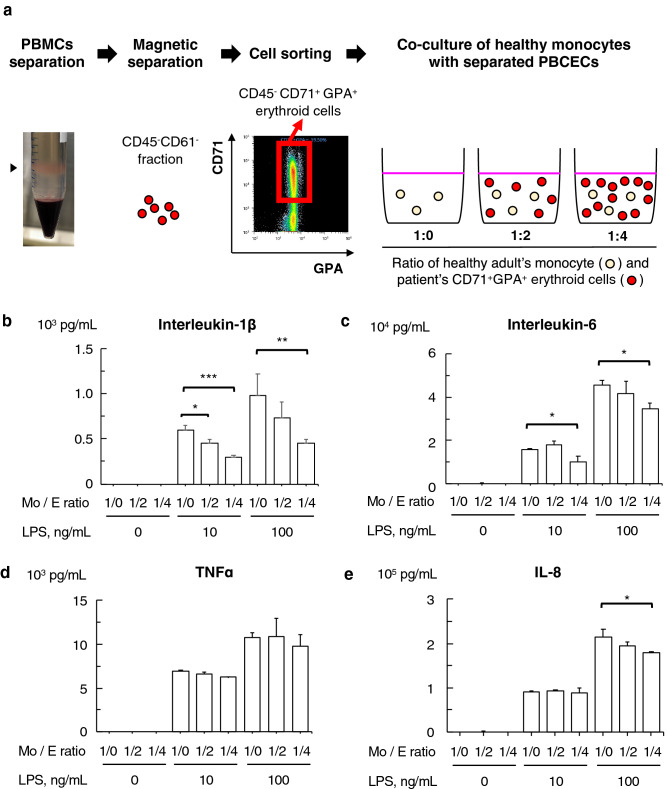
Suppression of cytokine release from monocytes by peripheral CD71^+^ erythroid cells derived from patients. (**a**) A schematic method describing the isolation of peripheral CD71^+^ erythroid cells and the co-culture of monocytes with erythroid cells. Peripheral CD45^−^ CD71^+^ GPA^+^ erythroid cells were isolated from peripheral mononuclear cells of SoJIA patients using magnetic separation and a cell sorting technique. Cells were co-cultured in three ratio patterns of patient’s CD71^+^ erythroid cells to heathy adult’s monocytes. (**b**–**e**) The levels of (**b**) interleukin-1β, (**c**) interleukin-6, (**d**) tumor necrosis factor-α, and (**e**) interleukin-8 in the culture supernatant after stimulation with lipopolysaccharide at 0, 10, and 100 ng/mL. Monocytes from a healthy control were co-cultured with peripheral CD71^+^ erythroid cells derived from a SoJIA patient at ratios of 1:0, 1:2, and 1:4. The experiment was repeated three times using PBCECs derived from three different patients, and each culture was performed in triplicate. Data are representative of three independent experiments. Data are represented as the mean ± SD and analyzed by the Mann–Whitney *U* test with Bonferroni correction. *P < 0.05; **P < 0.01; ***P < 0.001. *LPS* lipopolysaccharide, *Mo: E ratio* mixture ratio of healthy monocytes and the patient-derived peripheral CD71^+^ erythroid cells.

Human erythroid cells in umbilical cord blood have immunoregulatory effects^1,13^. To determine the immunosuppressive function of SoJIA-driven PBCECs, we investigated the monocyte responsiveness to lipopolysaccharide (LPS) in the presence or absence of PBCECs obtained from patients with SoJIA. We focused on the effect of CECs on monocytes but not lymphocytes, as SoJIA is an autoinflammatory disease involving excessive hypercytokinemia triggered by the activation of the innate immune system^35,36^. PBCECs were procured in combination with magnetic microbeads and fluorescent cell sorting from patient’s PBMCs to obtain a > 96% purity population (Fig. 4a and Supplementary Fig. S3 online). CD14^+^ monocytes were collected from PBMCs of healthy donors by microbeads. The co-culture of healthy monocytes and patients’ PBCECs was performed in 1:0, 1:2, and 1:4 ratios (Fig. 4a). The concentration of inflammatory cytokines was measured 18 h after LPS stimulation. The co-culture significantly suppressed the production of IL-1β, IL-6, and IL-8 but not TNFα, and this effect was enhanced in a ratio-dependent manner (Fig. 4b–e).

### The high expression of ARG2 and the regulatory potential in SoJIA-driven PBCECs

**Figure 5:**
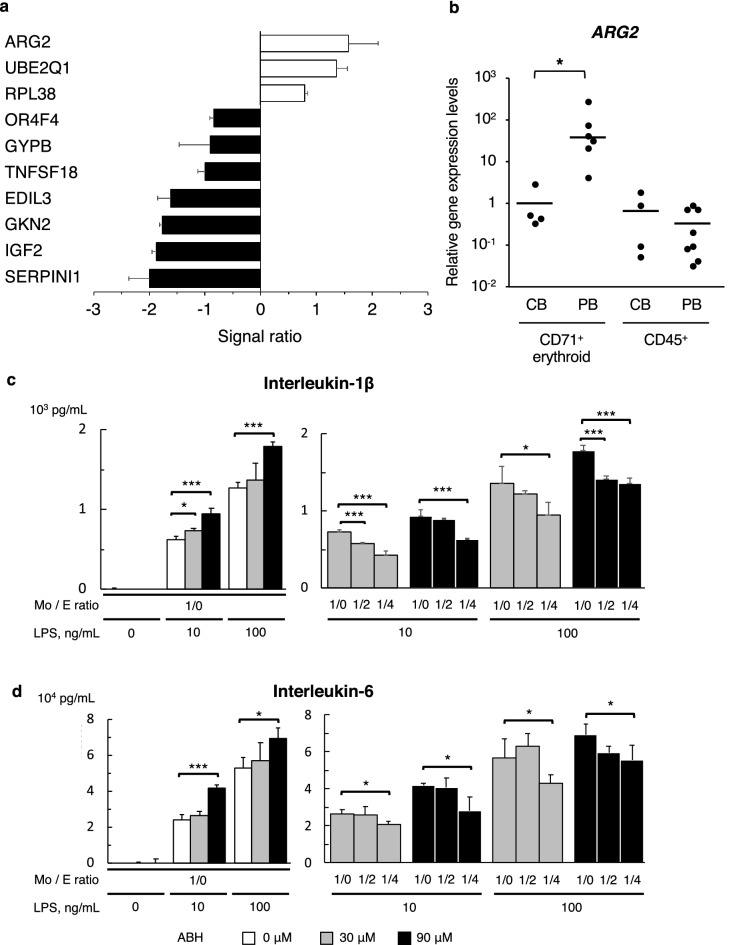
Production of inflammatory cytokines induced by arginase inhibitor diminished by co-culture with peripheral CD71^+^ erythroid cells derived from SoJIA patient. (**a**) Upregulated and downregulated genes in PBCECs compared to control neonatal cord blood. (**b**) Gene expression of *ARG2* in CD71^+^ erythroid cells and CD45^+^ lymphocytes and monocytes extracted from mononuclear cells in peripheral blood of SoJIA patients and neonatal cord blood. (**c**, **d**) The levels of (**c**) interleukin-1β and (**d**) interleukin-6 in the culture supernatant after stimulation with lipopolysaccharide at 0, 10, and 100 ng/mL. Healthy donor monocytes are cultured without (left graph) or with PBCECs obtained from SoJIA patients at ratios of 1:0, 1:2, and 1:4 (right graph). ABH was added to the culture medium at 0, 30, and 90 μM. The experiment was repeated three times using PBCECs derived from three different patients, and each culture was performed in triplicate. Data are representative of three independent experiments. Data were represented as the mean ± SD and analyzed by the Mann–Whitney *U* test with Bonferroni correction. *P < 0.05; **P < 0.01; ***P < 0.001. *CB* cord blood, *PB* peripheral blood, *LPS* lipopolysaccharide, *Mo: E ratio* mixture ratio of healthy monocytes and patient-derived peripheral CD71^+^ erythroid cells, *ABH* amino-2-borono-6-hexanoic acid.

To further assess the molecular mechanism underlying the effect in PBCECs, we compared the gene expression profile of PBCECs with that of CECs obtained from umbilical cord blood of healthy donors. The gene with the highest expression in PBCECs from SoJIA patients was *ARG2* (Fig. 5a), which is a key player responsible for the immunosuppressive function of circulating CECs in newborn mice^1^. Quantitative PCR confirmed that the amount of *ARG2* transcripts in SoJIA-driven PBCECs was higher than that in CECs from umbilical cord blood or CD45^+^ cells from umbilical cord blood and the peripheral blood of healthy donors (Fig. 5b).

We then investigated the cytokine release from LPS-stimulated monocytes in co-culture with PBCECs in the presence of the arginase inhibitor, ABH. The presence of ABH led to the increased production of each cytokine (Fig. 5c,d and Supplementary Fig. S4a,b online), although the immunosuppressive effect of PBCECs on the production of IL-1β, IL-6, and IL-8 was not canceled after the addition of ABH (Fig. 5c,d and Supplementary Fig. S4a,b online). These results suggested that the immunosuppressive function of PBCECs could be driven by not only arginase-2 but also other molecules.

### Galectin-3 and IL-18 binding protein do not mediate the regulatory effect of PBCECs

To identify other soluble immunomodulators from CECs, we focused on galectin-3 and IL-18BP. A recent study revealed the high gene expression of galectin in CECs from neonatal mice^10^. Among the galectin family proteins, we studied galectin-3 because it showed the highest expression of all galectins in human CECs according to the BioGPS database. IL-18BP was also targeted because recombinant IL-18BP effectively controlled the disease activity of SoJIA^37^.

However, co-culture with PBCECs did not increase the levels of galectin-3 or IL-18BP in culture supernatants (Supplementary Fig. S4c,d online). These findings indicate the involvement of other molecules or direct cell–cell interaction in the immunosuppressive property of PBCECs obtained from SoJIA patients.

## Discussion

We demonstrated for the first time an increased number of PBCECs in patients with systemic inflammatory disease. The number of circulating CECs in SoJIA was the largest among febrile diseases and decreased after disease control was achieved. The PBCEC counts were closely correlated with several markers related to erythropoiesis and inflammation. Among these, IL-18, sTNFR1, and hepcidin levels were significantly higher in patients with SoJIA than in those with KD or oligo/polyarticular SoJIA. SoJIA-driven PBCECs suppressed IL-1β, IL-6, and IL-8 production by monocytes of healthy controls. Despite the *ARG2* expression being higher in SoJIA-PBCECs than in umbilical cord blood CECs, SoJIA-PBCECs showed an arginase-independent cytokine-producing ability after LPS stimulation. These results suggest that human PBCECs are driven to the periphery to exert a counteracting effect on the progression of non-infectious systemic inflammation.

Erythropoiesis is involved in the development of inflammation. In the initial phase of inflammation, myeloid cells proliferate in the bone marrow, and inflammatory cytokines are produced from innate immune cells in response to pathogen-associated molecular patterns (PAMPs) and damage-associated molecular patterns (DAMPs)^38^. TNFα inhibits the proliferation of erythroid precursor cells, and IFNγ-activated macrophages shorten the lifespan of erythrocytes by phagocytosis. IL-6 modulates both the intestinal absorption and the iron release from macrophages through hepcidin, thus leading to hypoferremia and hyperferritinemia, which diminish the number of erythroid precursors. Inflammation therefore usually decreases the survival and production of erythrocytes.

In contrast, pregnancy, infection, hypoxia, and acute anemia itself induce erythropoiesis. Stress erythropoiesis resembles fetal erythropoiesis in many ways^39^. In a murine model of stress erythropoiesis, monocytes were recruited to the spleen and formed an erythropoiesis niche, called erythroblastic islands, with erythroid progenitors^40,41^. Splenic erythroid progenitors share surface markers with burst-forming unit-erythroid progenitors (BFU-Es) from murine fetal liver^42^. Increased serum erythropoietin levels trigger the transition of erythroid progenitors from proliferation to differentiation^43^. Erythropoietin-dependent signaling in macrophages induces prostaglandin E2 to promote the differentiation of erythroid precursors^44^. Unbalanced hematopoiesis causes erythropoiesis to occur outside of the bone marrow in the spleen and liver. In this line, stress erythropoiesis may be a better reflection of this phenomenon than extramedullary erythropoiesis^44,45^.

The expansion of the erythroid compartment is induced into the spleen of mice infected with *Salmonella* or murine *Plasmodium*^46,47^. Toll-like receptor (TLR)-2 mediated inflammation also led to stress erythropoiesis through Myd88-dependent signaling^48,49^, suggesting the involvement of innate immune signaling. In this line, the expansion of PBCECs in SoJIA and KD, pediatric autoinflammatory diseases deeply involved in the innate immune response^35,50^, may reflect this stress erythropoiesis. The positive correlation between erythropoietin levels and the amount of PBCECs also suggests the potential contribution of stress erythropoiesis to the expansion of CECs in autoinflammatory diseases. Several studies have reported that the expansion of PBCECs was related to a high mortality or disease severity^5,7–9^. Macrophage activation syndrome, an extremely severe manifestation of systemic inflammation with cytokine storm, occurs more frequently in SoJIA than in KD. This may be one of the reasons why SoJIA patients show an increased number and proportion of PBCECs compared with KD patients. Given the immunoregulatory function of PBCECs, they may be recruited to the periphery to suppress the excessive systemic inflammation.

TNFα and IL-1β are key cytokines that promote the proliferation and differentiation of erythroid progenitors in the spleen of mice^49^, although IFNγ does not affect the expansion of BFU-Es^48^. Thus, stress erythropoiesis develops in a setting of not only hypoxia but also infection and inflammation via innate immune signaling. We showed that serum levels of IL-18, a member of the IL-1 family, and sTNFR1, which reflects TNFα activity, were markedly increased in SoJIA patients and positively correlated with the PBCEC count. These results indicate the association between stress erythropoiesis and an increase in PBCECs. Excessive inflammatory cytokines derived from activated macrophages may augment the production of PBCECs. In contrast, Dunsmore et al.^51^ demonstrated a reduced frequency and impaired function of CD45^+^ CD71^+^ erythroid cells in pregnant women with inflammatory bowel disease. We hypothesized three reasons why PBCECs decreased despite their chronic inflammatory condition, in contrast to the findings in our cohort. First, their PBCECs were considered to have been induced mainly by pregnancy, but not inflammation, as PBCECs were observed even in healthy pregnant women. Second, most of the participants were well-controlled with treatment such as TNF inhibitors, while we focused on the acute-phase patients. Third, the mechanism underlying the production might differ between CD45^+^ and CD45^−^ CECs. Given the differences in the results, further studies are needed to evaluate the factors responsible for these differences.

In the present study, we were unable to obtain direct evidence of the involvement of arginase-2 in the immunosuppressive effect of PBCECs. The increased expression of *ARG2* in PBCECs compared with CECs from cord blood suggests the involvement of arginase-2 in the immunoregulatory function of PBCECs. However, arginase inhibition did not cancel the suppression of cytokine secretion from monocytes. Comparing these CECs may not be a suitable way of assessing ARG2, as cord blood CECs have limited potential for gene expression^13^. Recently, Namdar et al.^15^ detected increased numbers of PBCECs in HIV patients, and these cells enhanced HIV infection and allowed the virus to replicate in CD4^+^ T cells, indicating that PBCECs control inflammation but not infection. However, they did not detect any direct association of either arginase-2 or transforming growth factor β. To identify molecules related to the immunosuppressive effect of PBCECs, we focused on galectin-3 and IL-18BP but were unable to determine their involvement. Further studies are needed to determine the causality and molecular mechanism underlying the mobilization of PBCECs in inflammatory disorders. The latest research has demonstrated that CECs modulate different immune cells via other soluble mediators such as TGF-β and ROS or through cell–cell interactions (e.g. PDL-1, VISTA)^6,10,14,45^. These investigations offer useful directions for our future research.

SoJIA and KD share characteristics of an ongoing high fever, skin rash, and occasional arthritis during the active febrile phase. The distinct proportion of PBCECs may be a useful marker for the differential diagnosis of SoJIA and KD. Alternatively, the expansion of PBCECs may be induced by SoJIA-specific factors and involve the activated pathway of immune signaling.

Several limitations associated with the present study warrant mention. First, the assay was individually performed at the time of the diagnosis but at different febrile days of illness. Patients might thus have had clinical heterogeneity, even when they had the same disease, based on the diagnostic criteria. Second, dead cell contamination and erythrocyte aggregation tend to occur in patients with inflammatory diseases, although only fresh samples obtained just after blood sampling were used in this study. Third, the equal proportion (1:1) of monocytes and CECs might recapitulate the in vivo reaction in patients with SoJIA, as the median proportions of monocytes and CECs in PBMCs were 9.5% (range 2.0–28.9%) and 8.2% (range 2.4–24.2%), respectively.

In conclusion, we demonstrated the expansion of PBCECs in pediatric inflammatory diseases, especially SoJIA. PBCECs collected in the acute febrile phase of SoJIA showed an immunoregulatory function not only via arginase-2. Further studies concerning stress hematopoiesis will help clarify the biological role of primitive red blood cells in the self-defense system of mammals.

## Methods

### Human subjects

Patients who received a diagnosis of and treatment for febrile inflammatory diseases at the Kyushu University Hospital were enrolled in the study. For real-time quantitative polymerase chain reaction (PCR) in Fig. 1, we used the cDNA extracted from PBMCs of patients with SoJIA (n = 21), KD (n = 10), bacteremia (n = 8), oligo/polyarticular JIA (n = 4), and systemic lupus erythematosus (n = 5) as well as healthy controls (n = 27), which had been obtained for our previous study^32^. The median age (range) was 6 (2–14), 5 (1–7), 5 (0–9), 14 (13–16), 13 (10–16), and 20 (5–33) years old, respectively. The percentages of males were 45%, 60%, 50%, 25%, 20%, and 44%, respectively. For the flow cytometry analysis shown in Figs. 2a and 3a, the complete blood count test in Fig. 3b–d, and serum assay in Table 2, blood samples were newly collected from the patients with SoJIA (n = 12), KD (n = 21), and oligo/polyarticular JIA (n = 11) in the acute phase of disease, as well as healthy controls (n = 6), after informed consent had been obtained. The clinical data of these patients are shown in Table 1. Paired samples obtained from some of the patients in the acute and convalescent phases of their diseases were used for assays in Fig. 3a,d. Acute phase samples from some patients with SoJIA were also used for assays in Figs. 4 and 5. For the microarray analysis in Fig. 5, human umbilical cord blood samples were obtained from healthy full-term pregnant women with informed consent at the Kyushu University Hospital.

### The diagnosis and blood sampling

We used the International League of Associations for Rheumatology criteria for the diagnosis of JIA^16^ and the diagnostic criteria of the Japanese KD Research Committee for the diagnosis of KD^52^. Complete KD was only included in cases with at least five of six principal symptoms or four principal symptoms accompanied by coronary artery abnormalities. All patients were diagnosed by trained pediatric rheumatologists and cardiologists based on the clinical manifestations and laboratory findings.

Venous blood samples (3 mL) were obtained only when there was a clinical need for blood tests. PBMCs were isolated immediately by density gradient centrifugation using Lymphocyte Separation Medium (MP Biomedicals, Santa Ana, CA, USA).

### Total RNA extraction

Total RNA was extracted from PBMCs using an RNeasy Mini Kit (Qiagen, Germantown, Germany), according to the manufacturer’s instructions. The quantity and quality of RNA were determined by an ND-1000 spectrophotometer (NanoDrop Technologies, Wilmington, DE, USA) and a 2200 TapeStation (Agilent Technologies, Santa Clara, CA, USA), respectively.

### The microarray analysis and filter criteria

We used the gene expression data obtained in our previous study to compare the gene expression profiles of PBMCs in Supplementary Table S1 online^32^. For the microarray analysis of PBCECs, the cRNA was amplified, labeled with total RNA using GeneChip^®^ WT Pico Kit (Thermo Fisher Scientific, Waltham, MA, USA), and hybridized to a Clariom™ D Assay, human (Thermo Fisher Scientific), according to the manufacturer's instructions. All hybridized microarrays were scanned by an Affymetrix scanner.

Relative hybridization intensities and background hybridization values were calculated using the Affymetrix Expression Console™ (Thermo Fisher Scientific). The raw signal intensities of all samples were normalized with the SST-RMA algorithm (gene level) using the Affymetrix Expression Console 1.4.1 software program (Thermo Fisher Scientific). To identify up- and down-regulated genes, we calculated the Z-scores and ratios (non-log scaled fold-change) from the normalized signal intensities of each probe for a comparison between control and experimental samples. We then established criteria for regulated genes, as follows: up-regulated genes, Z-score ≥ 2.0 and ratio ≥ 1.5-fold; down-regulated genes, Z-score ≤ − 2.0 and ratio ≤ 0.66. Microarray data of PBCECs are available at Gene Expression Omnibus under accession number GSE164120.

The preparation of the samples, microarray hybridizations, and bioinformatics analysis were performed by Cell Innovator at Kyushu University (Fukuoka, Japan, http://www.cell-innovator.com/).

### Real-time quantitative PCR

Double-stranded complementary DNA was synthesized from the total RNA using a high-capacity RNA-to-cDNA kit (Applied Biosystems, Foster City, CA, USA). The *AHSP*, *HBD*, and *CA1* mRNA expression was measured using Taqman^®^ Gene Expression Master Mix (Thermo Fisher Scientific) and TaqMan^®^ gene expression assays Hs00372339_g1, Hs00426283_m1, and Hs00266139_m1 (Thermo Fisher Scientific), respectively. The ARG2 mRNA levels were measured using the SYBR^®^ Green PCR Master Mix (Applied Biosystems). The sequences of *ARG2*-specific primers were (forward) AAGCTGGCTTGATGAAAAGGC and (reverse) GCGTGGATTCACTATCAGGTTGT, as described in the previous study^13^. Each reaction was carried out using an ABI Plus One Real-Time PCR system (Applied Biosystems). The expression data for each gene were normalized against β-actin. The data were analyzed using the ddCt method.

### Flow cytometry

Fresh PBMCs were used for each flow cytometry analysis. Cells were suspended in phosphate-buffered saline (PBS) and incubated with antibody cocktails in the dark at room temperature for 15 min. After incubation, the cells were washed and resuspended in PBS and then analyzed. Data were acquired using an EC-800 (Sony Biotechnology Inc., Champaign, IL, USA) and analyzed with the Kaluza software program (Beckman Coulter, Brea, CA, USA). Forward scatter and side scatter gates were set to capture the cell population of interest, including monocytes and lymphocytes. We excluded the debris fractions to eliminate aberrant binding events due to dead cells as other groups did^34,51^. The following antibodies were used: anti-GPA (17-9987-42; eBiosciences, San Diego, CA, USA), anti-CD36 (IM0766U; Beckman Coulter), anti-CD45 (2120080; Sony Biotechnology Inc.), and anti-CD71 (334106; Biolegend, San Diego, CA, USA).

### Isolation of monocyte and PBCECs from human PBMCs

Monocytes were isolated from PBMCs obtained as above with positive magnetic sorting techniques, using anti-CD14 monoclonal antibody (mAb)-conjugated microbeads (Miltenyi Biotec, Bergisch Gladbach, Germany). Erythroid cells were isolated from PBMCs with negative magnetic sorting techniques, using anti-CD45 and CD61 microbeads (Miltenyi Biotec). CD71^+^ GPA^+^ erythroid cells were then extracted with an SH800 Cell Sorter (Sony Biotechnology Inc.).

### Co-culture of monocytes with PBCECs

The purified CD14^+^ monocytes were cultured in RPMI-1640 culture medium (Gibco Laboratories, Grand Island, NY, USA) supplemented with 10% heat-inactivated fetal calf serum and 1% gentamicin in a 96-well plate at 5 × 10^4^ cells/well. The purified PBCECs were added at 0, 1 × 10^5^, and 2 × 10^5^ cells/well. In some experiments, cells were treated 1 h before and during co-culture with the arginase inhibitor amino-2-borono-6-hexanoic acid ABH (Cayman Chemical, Ann Arbor, MI, USA) at concentrations of 30 and 90 mM. Cells were stimulated with lipopolysaccharide (LPS) (Sigma) at concentrations of 0, 10, and 100 ng/mL, and the culture supernatants were collected after 18 h.

### Cytokine measurements

Interleukin (IL)-1β, IL-6, IL-8, tumor necrosis factor (TNF)α, soluble TNF receptors, interferon-gamma (IFN-γ), granulocyte colony-stimulating factor (G-CSF), and granulocyte macrophage colony-stimulating factor (GM-CSF) in the serum and culture supernatants were measured using the Cytometric Bead Array System (BD Biosciences, San Jose, CA, USA), according to the manufacturer’s instructions. The concentration of each cytokine was acquired on the EC-800 and analyzed using the FCAP Array software program (BD Biosciences). The following enzyme-linked immunoassay kits were used: erythropoietin (BMS2035; R&D Systems, Minneapolis, MN, USA), hepcidin (DHP250; R&D systems), IL-18 (BMS267INST; Thermo Fisher Scientific), IL-18 binding protein (IL-18BP) (EHIL18BP; Thermo Fisher Scientific), and galectin-3 (DGAL30; R&D systems).

### Statistical analyses

We used a chi-squared test to compare the proportions of categorical variables and the Mann–Whitney *U* test to compare the medians of continuous variables. Multiple comparisons were performed with Bonferroni correction. A *P* value < 0.05 was considered statistically significant for all experiments. All data were analyzed using the JMP Pro 15.1.0 software program (SAS Institute Inc., Cary, NC, USA; http://jmp.com).

### Study approval

This study was conducted in accordance with the Declaration of Helsinki. The Institutional Review Board of Kyushu University approved the study (#388-02). Written informed consent was received from all participants prior to their inclusion in the study.

## Supplementary Information

Supplementary Information.

## Data Availability

All data generated or analyzed during this study are included in this article and the supplementary information files.
