# Supplementary material for: The immunoregulatory function of peripheral blood CD71+ erythroid cells in systemic-onset juvenile idiopathic arthritis
Source: Sci Rep. 2021 Jul 13;11:14396. doi: 10.1038/s41598-021-93831-3 (PMC8277864; doi:10.1038/s41598-021-93831-3)
Supplement: Supplementary file 1 — Supplementary Information. [file 41598_2021_93831_MOESM1_ESM.pdf]

Supplementary Fig. S1

a

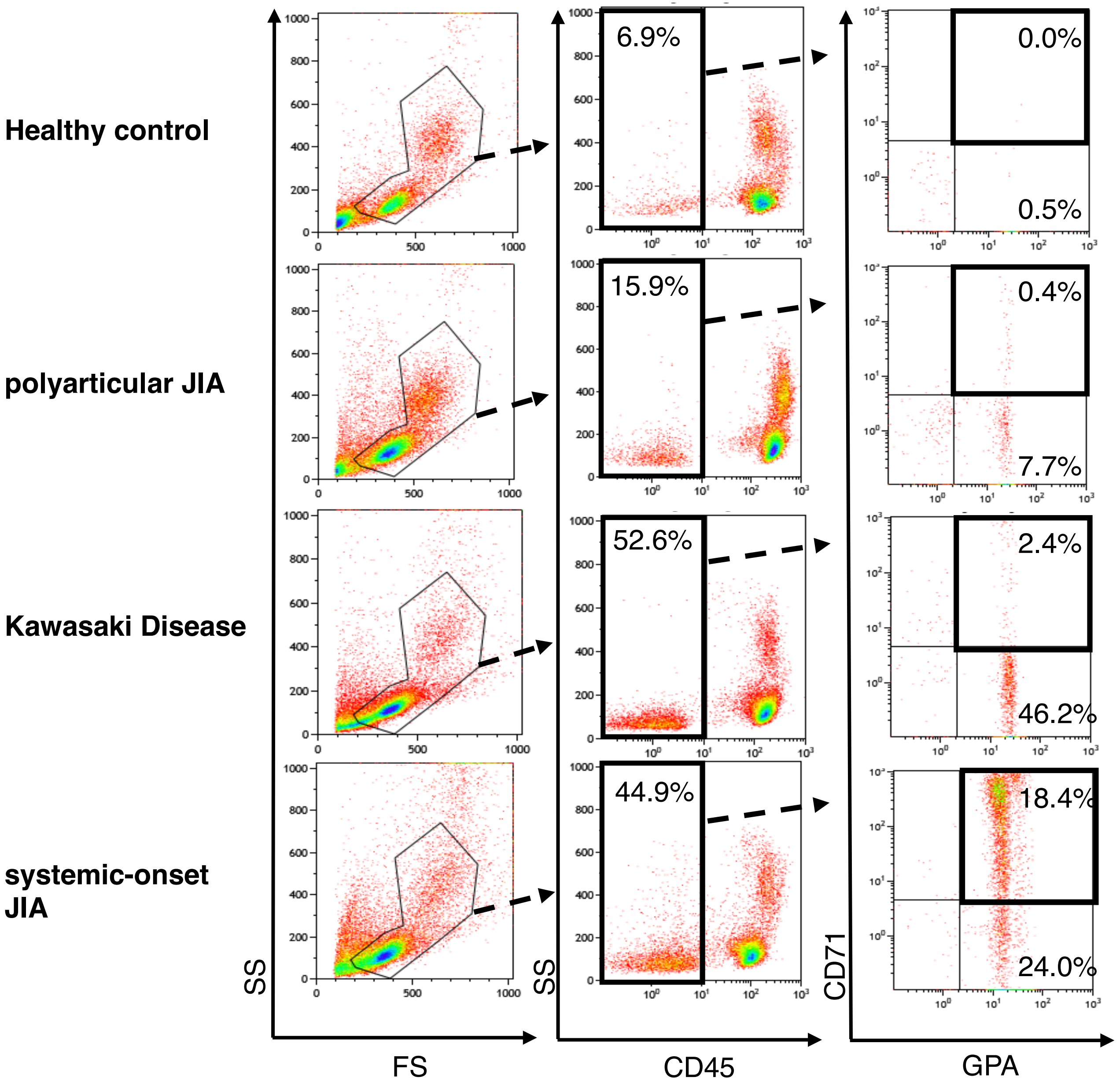

Supplementary Fig. S1a. Identification of CD45$^-$CD71$^+$GPA$^+$ erythroid cells in peripheral blood mononuclear cells by flow cytometry. Representative data of a healthy control and each patient with polyarticular JIA, Kawasaki disease, or systemic-onset JIA. The number on the plots was the percentage in the mononuclear cell region. Abbreviations: JIA, juvenile idiopathic arthritis; FS, Forward scatter; SS, side scatter; GPA, Glycophorin A (CD235a).

Supplementary Fig. S1

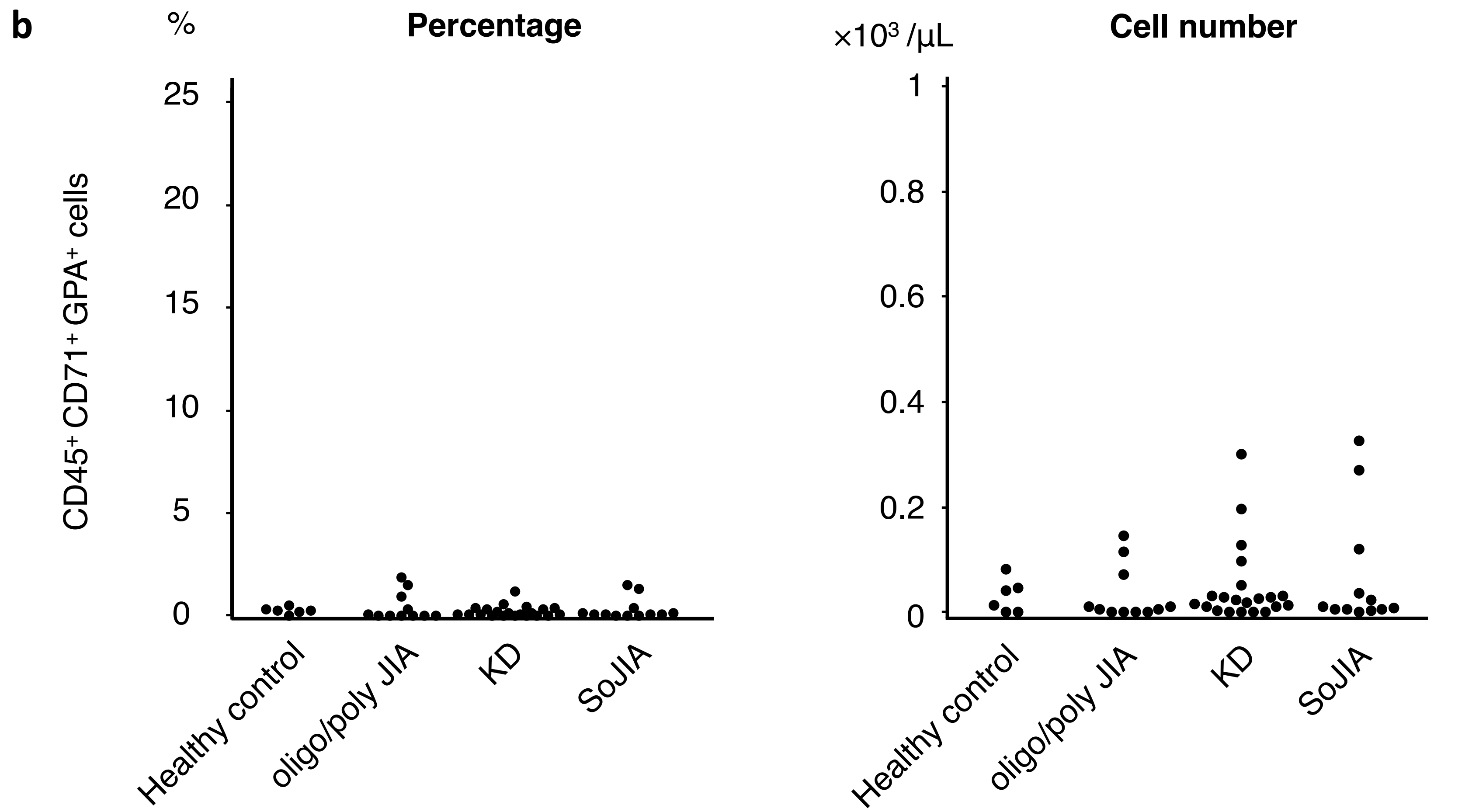

Supplementary Fig. S1b. The percentage and absolute number of $CD45^+CD71^+GPA^+$ erythroid cells in peripheral blood mononuclear cells among healthy control and patients with oligo/polyarticular JIA, KD, and SoJIA. Abbreviations: oligo/poly JIA; oligo/polyarticular juvenile idiopathic arthritis; KD, Kawasaki disease; SoJIA, systemic-onset juvenile idiopathic arthritis.

Supplementary Fig. S1

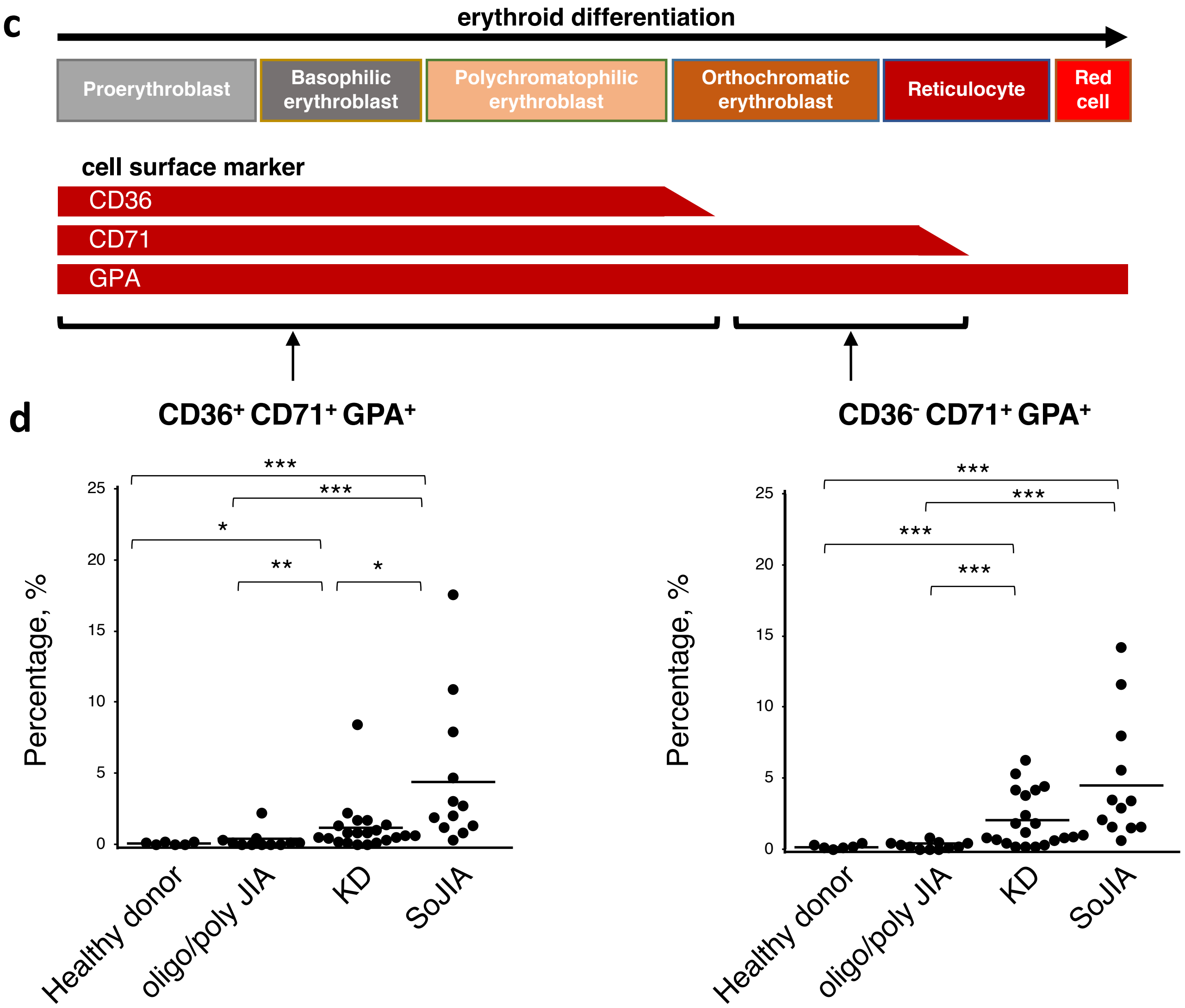

Supplementary Fig. S1c-d. (c) Schema of the transition of surface markers on erythroid precursor cells, CD36, CD71 and GPA, in erythroid differentiation. (d) The percentage of $CD45^{-}CD36^{+}CD71^{+}GPA^{+}$ early erythroid cells and $CD45^{-}CD36^{-}CD71^{+}GPA^{+}$ late erythroid cells in peripheral blood mononuclear cells among healthy control and patients with oligo/polyarticular JIA, KD, or SoJIA. Horizontal bar means average. Data are analyzed by the Mann-Whitney *U* test with the Bonferroni correction. **P* <0.05; ***P* <0.01; ****P* <0.001. Abbreviations: oligo/poly JIA, oligo/polyarticular juvenile idiopathic arthritis; KD, Kawasaki disease; SoJIA, systemic-onset juvenile idiopathic arthritis; GPA, Glycophorin A (CD235a).

Supplementary Fig. S2

	WBC	CRP	Hb	RDW	Reticulocyte	Ferritin	Erythropoietin	Hepcidine	IL-18	IL-6	Galectin3	IL-18BP	sTNFR1	sTNFR2	IFNg	G-CSF	GM-CSF	Number of PBCEC	Percentage of PBCEC
Duration of fever *	-0.23	0.02	**-0.56**	-0.04	-0.27	0.13	-0.13	0.34	**0.53**	-0.27	-0.09	0.15	0.02	**0.46**	0.07	-0.15	0.13	-0.17	-0.12
	0.28	0.92	**0.01**	0.87	0.25	0.56	0.70	0.23	**0.01**	0.22	0.69	0.51	0.94	**0.03**	0.74	0.48	0.56	0.44	0.58
WBC **		**0.67**	-0.09	**0.43**	0.22	**0.38**	**0.71**	**0.60**	0.24	**0.61**	**0.56**	-0.05	**0.67**	0.21	-0.30	0.16	-0.24	**0.49**	**0.61**
		**<.001**	0.63	**0.02**	0.25	**0.03**	**0.005**	**0.007**	0.18	**<.001**	**<.001**	0.80	**<.001**	0.24	0.09	0.36	0.19	**0.004**	**<.001**
CRP **			-0.30	0.00	-0.19	**0.80**	**0.85**	**0.67**	**0.61**	**0.42**	**0.66**	0.19	**0.66**	**0.41**	-0.08	0.22	-0.16	**0.38**	**0.55**
			0.10	0.98	0.33	**<.001**	**<.001**	**0.002**	**<.001**	**0.02**	**<.001**	0.28	**<.001**	**0.02**	0.65	0.21	0.38	**0.03**	**<.001**
Hb **				0.06	0.15	-0.14	-0.33	-0.32	-0.21	0.25	0.06	-0.15	-0.19	**-0.45**	-0.04	0.00	-0.04	-0.05	-0.07
				0.74	0.42	0.43	0.25	0.18	0.24	0.17	0.75	0.41	0.30	**0.009**	0.81	1.00	0.80	0.76	0.70
RDW **					**0.42**	-0.10	-0.13	-0.31	0.08	**0.46**	0.13	-0.10	0.15	-0.14	-0.10	0.10	-0.07	**0.56**	**0.45**
					**0.03**	0.60	0.66	0.20	0.68	**0.01**	0.47	0.58	0.41	0.46	0.59	0.60	0.69	**0.0011**	**0.01**
Reticulocyte ***						-0.20	0.01	**-0.52**	-0.19	-0.06	0.06	-0.29	-0.08	-0.24	-0.24	-0.05	-0.11	0.12	0.05
						0.31	0.97	**0.03**	0.33	0.75	0.75	0.13	0.66	0.21	0.21	0.80	0.58	0.54	0.81
Ferritin **							**0.88**	**0.52**	**0.68**	**0.40**	**0.72**	0.19	**0.53**	0.24	-0.01	0.07	-0.15	0.02	0.22
							**<.001**	**0.02**	**<.001**	**0.02**	**<.001**	0.29	**0.002**	0.17	0.94	0.69	0.42	0.90	0.21
Erythropoietin ****								0.53	**0.59**	**0.91**	**0.82**	0.07	**0.66**	0.27	-0.17	0.29	-0.29	**0.55**	**0.67**
								0.054	**0.03**	**<.001**	**<.001**	0.81	**0.01**	0.34	0.56	0.32	0.31	**0.04**	**0.009**
Hepcidine ****									**0.66**	**0.52**	**0.54**	**0.46**	**0.80**	**0.83**	0.05	0.14	-0.27	**0.68**	**0.64**
									**0.002**	**0.03**	**0.02**	**0.046**	**<.001**	**<.001**	0.83	0.56	0.26	**0.0012**	**0.003**
IL-18 **										**0.38**	**0.56**	0.24	**0.44**	**0.48**	-0.01	0.27	-0.01	0.29	**0.42**
										**0.03**	**<.001**	0.18	**0.0098**	**0.0046**	0.97	0.13	0.94	0.10	**0.02**
IL-6 **											**0.45**	0.02	**0.43**	0.09	-0.07	**0.58**	0.00	**0.50**	**0.55**
											**0.009**	0.92	**0.01**	0.61	0.70	**<.001**	0.98	**0.003**	**<.001**
Galectin3 **												0.28	**0.71**	0.30	0.06	0.03	-0.23	0.15	0.30
												0.11	**<.001**	0.08	0.75	0.85	0.19	0.39	0.08
IL-18BP **													**0.53**	**0.67**	**0.83**	-0.01	-0.12	0.11	0.13
													**0.0013**	**<.001**	**<.001**	0.98	0.49	0.54	0.46
sTNFR1 **														**0.61**	0.19	0.12	**-0.35**	0.26	**0.37**
														**<.001**	0.28	0.52	**0.042**	0.14	**0.03**
sTNFR2 **															**0.37**	0.26	0.01	0.17	0.24
															**0.03**	0.14	0.98	0.34	0.17
IFN-γ **																0.10	0.20	-0.02	-0.04
																0.59	0.25	0.91	0.80
G-CSF **																	**0.44**	**0.52**	**0.49**
																	**0.009**	**0.002**	**0.003**
GM-CSF **																		0.10	0.09
																		0.57	0.63
Number of PBCEC **																			**0.94**
																			**<.001**
Percentage of PBCEC **																			

Supplementary Fig. S2. Pearson's correlations matrix between the number or percentage of PBCEC and the continuous variables in patients with systemic-onset JIA, Kawasaki disease , or oligo/polyarticular JIA; * n = 0, 14, or 9, ** n = 10, 14 or 9; *** n = 9, 13, or 7; **** n = 3, 7, or 5. Upper and lower numbers in each matrix means Pearson's correlation coefficients (*r*) and *P* values. Shaded matrix indicates a significant association between two variables. Abbreviations: WBC, white blood cell; Hb, hemoglobin; IL-18BP, IL-18 binding protein; sTNFR, soluble TNF receptor; PBCEC, $CD71^{+}$ erythroid cells in peripheral blood; RDW, Red cell distribution width.

Supplementary Fig. S3

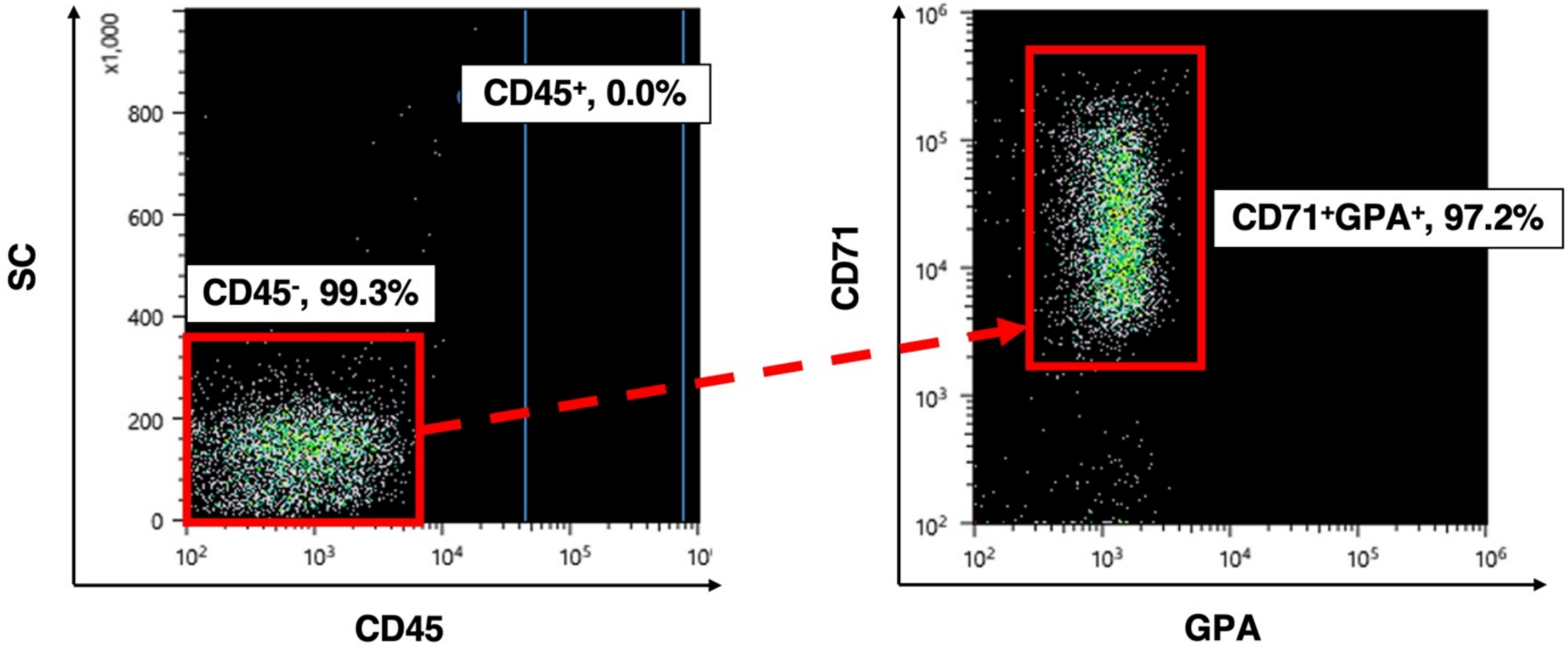

Supplementary Fig. S3. The purity of $CD45^{-}CD71^{+}GPA^{+}$ PBCECs sorted for the co-culture experiments.

Supplementary Fig. S4

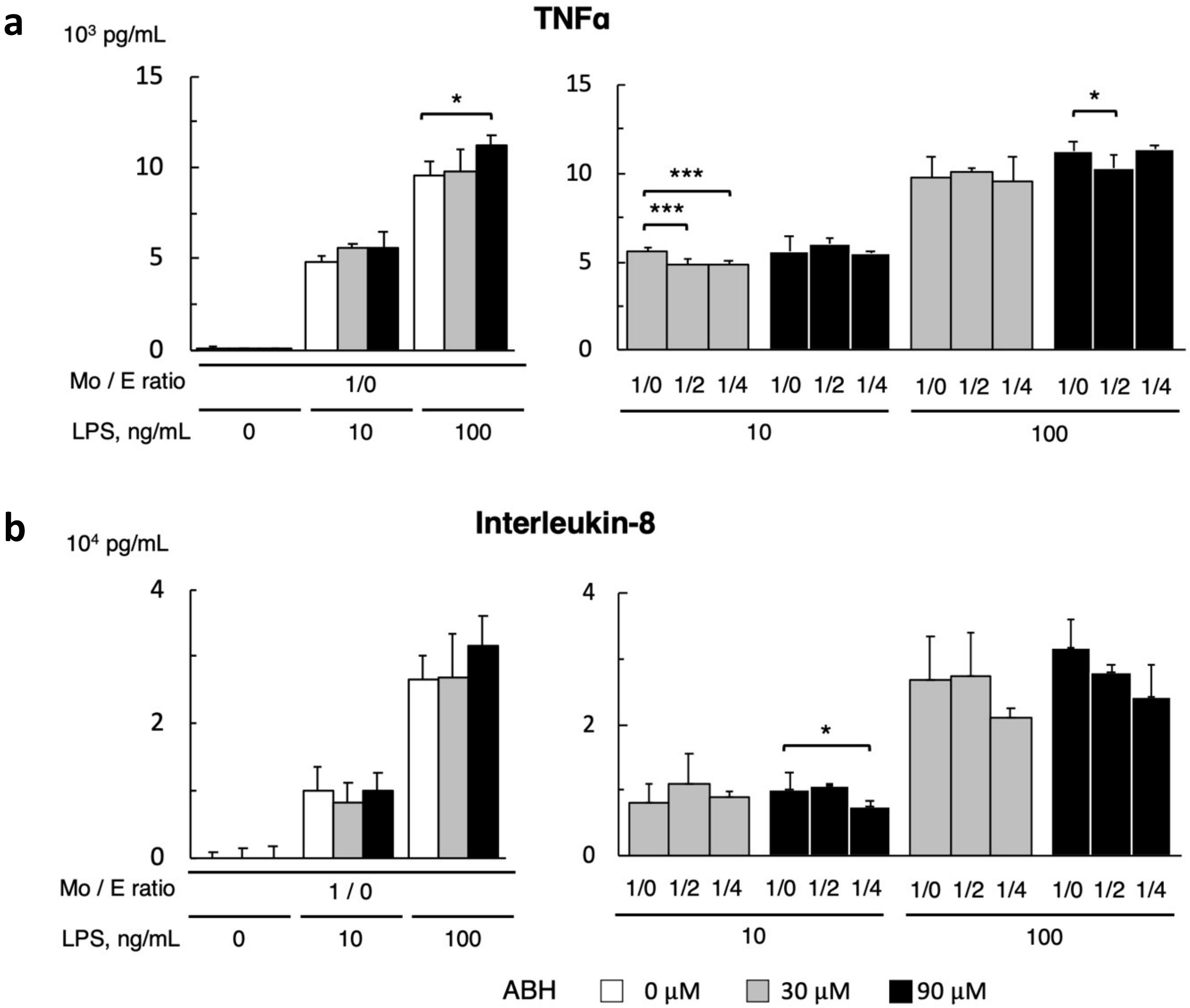

Supplementary Fig. S4a-b. The levels of (a) TNFα and (b) interleukin-8 in the culture supernatant after the stimulation with lipopolysaccharide at 0, 10, and 100 ng/mL. Culture of healthy monocytes was performed without (left graph) or with peripheral CD71+ erythroid cells derived from SoJIA patients at the ratio of 1:0, 1:2, and 1:4 (right graph). The arginase inhibitor, amino-2-borono-6-hexanoic acid (ABH), was added to the culture medium at 0, 30, and 90 μM.

Supplementary Fig. S4

c

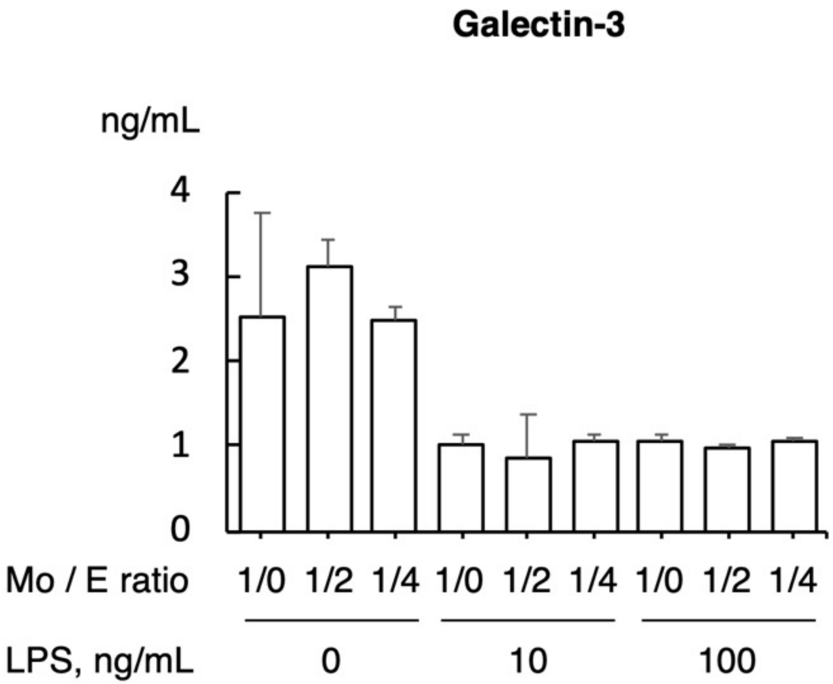

d

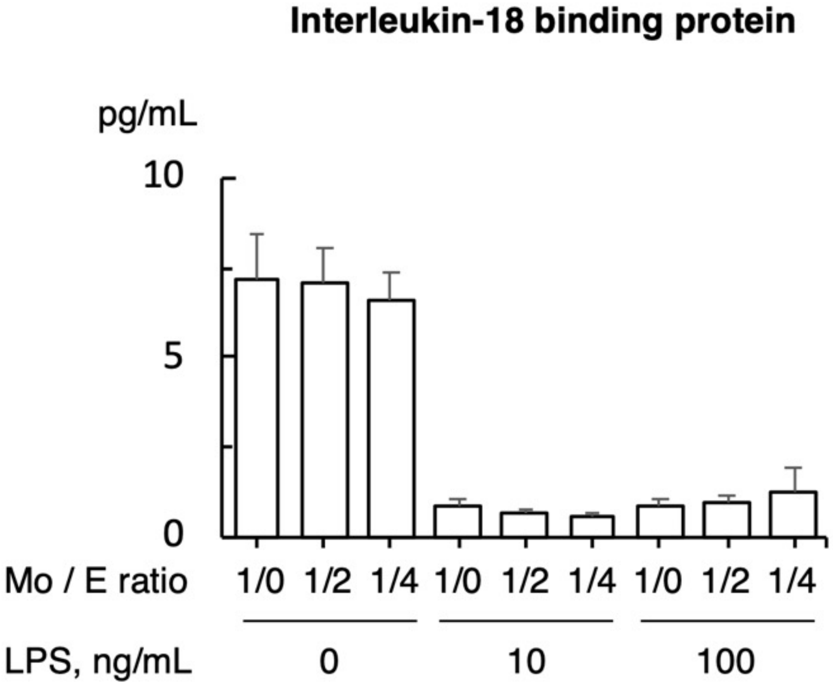

Supplementary Fig. S4c-d. The levels of (c) galectin-3 and (d) interleukin-18 binding protein in the culture supernatant after the stimulation with lipopolysaccharide at 0, 10, and 100 ng/mL. Monocytes from healthy controls were co-cultured with PBCECs at the ratio of 1:0, 1:2, and 1:4. The experiment was repeated three times using PBCECs derived from three different patients and each culture was performed in triplicate. Data are representative of three independent experiments. Data are represented as mean $\pm$ SD and analyzed by the Mann-Whitney *U* test with the Bonferroni correction. Error bars show standard error. Abbreviations: LPS, lipopolysaccharide; Mo: E ratio, the mixture ratio of healthy monocytes and patient-derived peripheral $CD71^{+}$ erythroid cells.

Supplementary Table S1

Gene symbol	Gene ID	Fold differences compared to a healthy control			Gene information
		SoJIA	KD	HNL	
AHSP	AGhsB210214	11.10	2.33	0.72	Erythroid associated factor
RAP1A	AGhsC010919	11.77	3.36	1.06	Homo sapiens mRNA for Raichu404X
HBD	AGhsA040815	8.24	4.08	0.96	Hemoglobin, delta
UROD	AGhsB021218	7.94	3.39	1.15	Uroporphyrinogen decarboxylase
MRPS7	AGhsA150924	7.19	2.97	1.09	Mitochondrial ribosomal protein S7
LRCH1	AGhsB021205	7.15	2.05	0.95	Leucine-rich repeats and calponin homology domain containing 1
CA1	AGhsA191622	6.48	2.56	0.55	Carbonic anhydrase I
ETS1	AGhsA081020	6.20	2.10	1.06	V-ets erythroblastosis virus E26 oncogene homolog 1
FGF7	AGhsC081320	7.01	1.53	1.07	Fibroblast growth factor 7
TCRDV1J1	AGhsC080416	5.87	2.35	0.94	Homo sapiens T-cell receptor delta chain
C19orf26	AGhsC010813	5.87	1.51	0.96	
C14orf131	AGhsA161624	5.71	1.54	1.29	Chromosome 14 open reading frame 131
VIPR2	AGhsA201419	4.91	1.85	1.02	Hypothetical protein LOC154822
TCN1	AGhsA180813	4.09	1.34	1.36	Transcobalamin I
SNCA	AGhsA020723	4.61	2.13	0.64	Synuclein, alpha
CAMP	AGhsA251203	3.59	0.83	1.17	Cathelicidin antimicrobial peptide
PRODH	AGhsC010804	4.46	1.79	1.06	Homo sapiens p53 induced protein
KIAA1853	AGhsC011216	4.59	1.37	0.99	KIAA1853 protein
SPTBN4	AGhsB050409	4.74	1.66	1.01	betaiv spectrin isoform sigma3
IMPA2	AGhsA141306	4.33	1.79	1.14	Inositol(myo)-1(or 4)-monophosphatase 2
EPB42	AGhsA160910	4.21	1.88	0.72	Erythrocyte membrane protein band 4.2
SASH1	AGhsB010122	3.92	1.81	1.05	
LR8	AGhsA201207	3.82	1.60	1.37	LR8 protein
TENS1	AGhsA051605	4.10	1.33	1.06	Tensin 3
DEFA3	AGhsA050803	2.74	1.02	0.23	Defensin, alpha 3, neutrophil-specific
MAOB	AGhsA120523	3.45	1.53	0.98	Monoamine oxidase B
OPRL1	AGhsA130505	3.48	1.46	0.92	Opiate receptor-like 1
FKSG39	AGhsB050117	3.63	1.37	0.87	Homo sapiens FKSG39
TPO	AGhsA110805	3.25	1.16	0.97	Thyroid peroxidase
C14orf87	AGhsB231409	2.89	1.38	1.35	Chromosome 14 open reading frame 87
CEAL1	AGhsB261113	3.22	1.18	0.97	Carcinoembryonic antigen-related cell adhesion molecule 19
AMT	AGhsA230817	3.01	1.22	1.00	Aminomethyltransferase
TM4SF9	AGhsB021520	2.76	0.98	0.85	transmembrane 4 superfamily
NOVA1	AGhsB150813	2.74	1.14	1.16	Neuro-oncological ventral antigen 1
REPIN1	AGhsA020604	3.09	1.05	1.13	Replication initiator 1
PEA15	AGhsA251414	2.28	1.07	0.97	Phosphoprotein enriched in astrocytes 15
EFHC2	AGhsA021015	2.57	0.81	0.89	Homo sapiens EF-hand domain (C-terminal) containing 2
C14orf159	AGhsB120516	2.37	0.97	0.98	Chromosome 14 open reading frame 159
STMN4	AGhsB261404	2.24	1.01	0.91	Stathmin-like 4

Upregulated genes with more than 2-fold difference in PBMCs obtained from patients with SoJIA (n=5) compared to those from patients with KD (n=3) and HNL (n=4) and a healthy donor. The data was obtained during our previous study.[27] HNL, histiocytic necrotizing lymphadenitis; KD, Kawasaki disease; SoJIA, systemic-onset juvenile idiopathic arthritis
